# A Clean Mouth Turneth Away Trouble

**Published:** 1911-06-15

**Authors:** Wm. W. Belcher

**Affiliations:** Rochester, N. Y.


### A CLEAN MOUTH TURNETH AWAY TROUBLE.

WM. W. BELCHER, D.D.S., ROCHESTER, N. Y.

A clean mouth is a safeguard against disease. A clean mouth turneth away trouble, is just as good a proverb, "as a soft answer turneth away wrath."

The full significance of these facts have not been understood by even the dental profession until a comparatively recent time. Dentists have been practicing preventive medicine but knew it not.

Not unlike many other great truths, the wonder is that we should not have recognized it earlier.

How delightfully simple. At least 75 per cent. of all our diseases are introduced through the mouth. If this be true, what scrupulous care should be taken to keep it clean and free from contagion.

As a matter of fact, how little care has been exercised by even the average reasonably neat person: they have given more attention to the cleanliness of their feet than this vestibule of life, the human mouth!

That a dirty mouth with its wealth of micro-organisms should spread disease and cause numberless ills seems quite simple; that such diseases as diphtheria, croup, and all respiratory troubles should be very much worse and many times caused by an unhygienic mouth seems equally true. That a child doubling his weight and at the school age assuming the added burden of his mental development, needs a good chewing equipment, and that it is absolutely essential to his future welfare seems, in the light of to-day, an undebatable truth. Why didn't we recognize this fact earlier?

A tuberculosis patient must have a good dental equipment if he is to make a successful fight against the "Great White Plague," and cannot recover if he be burdened with a filthy mouth harboring infection and uncleanliness, with the possibilities of constant reinfection to the human economy and the accompanying digestive troubles associated with this condition; and yet we have, in the past, practically neglected this important help. That the percentage of cures have been so low is not surprising, as that there should have been any at all.

How could we ever have expected a child with twelve to fifteen of its twenty teeth decayed, with exposed pulps, with teeth discharging their dirty slimy pus into the stomach and mixed with every mouthful of food, impairing the digestive fluids and burdening the body with poisonous toxins to keep up its studies at school or remain in good health?

Children in the past have survived these conditions; have lived in filth, and, in spite of these handicaps, have attained their mental and physical equipment; how did they ever do it? God only knows. How many of these children have been whipped and misunderstood at home, apologized for at school with a reputation for a bad temper, for being obstinate and dull, when the whole trouble was that they had to fight a diseased condition produced by a deficient and uncared for dental equipment with the poisoned and contaminated and poorly chewed food that refused to build healthy tissue and brain matter.

Can you wonder at this child with a diseased mouth and all its associated troubles, with adenoids, unable to keep his place in school, shunned by his companions and no place in their childish sports, becoming discouraged? He is told that he is not "bright," he loses faith in himself and looks with envy on those more favored. He plays "hookey" and finds congenial companions outside his school life and oft-times becomes a physical and moral degenerate.

Now we cannot escape this responsibility. The child of to-day is to be the ruler of to-morrow; the law maker and the governor of our large cities and the laws of our State are full of makeshifts placed there by those who did not have a "square deal" in our schools and suffered a mental handicap; they were never "free and equal" and this is part of the cost that every community is paying for poorly developed children.

Do you think this picture has been overdrawn? How many children whom you know have entered school full of promise and from some unknown cause have slowly drifted from bad to worse—lost their place and degenerated physically and mentally?

Dr. Gulick, after some time investigating this matter, says that two defective teeth in the mouth of a child will retard him for half a year in his studies.

What are we doing for these children in this land of ours? Why, practically nothing. We have plenty of money to fight doodle bugs, foot and mouth disease amoung our cattle and infectious diseases among our hogs and money for forest preserves and a Barge canal. Don't you think it would be economy to spend a little of this money on our greatest national asset, the boys and girls of to day who are so soon to be our rulers and lawmakers? How long, Oh Lord, how long shall the blind lead the blind.

These statements as here presented you may or may not believe, but the truth is strong and must prevail. Dentistry has a most important place in the practice of preventive medicine. You must accept this. Any other attitude in this year of our Lord is harking back to the Voodo doctor and the dark ages. Let in the light!—*The Dispensary Record.*

